# Hemophagocytic Lymphohistiocytosis Gene Variants in Severe COVID-19 Cytokine Storm Syndrome

**DOI:** 10.3390/v17081093

**Published:** 2025-08-08

**Authors:** Abhishek Kamath, Mingce Zhang, Devin M. Absher, Lesley E. Jackson, Walter Winn Chatham, Randy Q. Cron

**Affiliations:** 1 Heersink School of Medicine, University of Alabama at Birmingham (UAB), Birmingham, AL 35233, USA; akamath@uab.edu; 2 Division of Rheumatology, Children’s of Alabama, Birmingham, AL 35233, USA; mizhang@uabmc.edu; 3 Kaiser Permanente Bernard J. Tyson School of Medicine, Oakland, CA 94612, USA; devin.m.absher@kp.org; 4 Division of Clinical Immunology and Rheumatology, University of Alabama at Birmingham (UAB), Birmingham, AL 35233, USA; lejackson@uabmc.edu; 5 Division of Rheumatology, University of Nevada, Las Vegas (UNLV), Las Vegas, NV 89102, USA; w.chatham@unlv.edu

**Keywords:** COVID-19, cytokine storm syndrome, genetics, hemophagocytic lymphohistiocytosis, *DOCK8*, natural killer cell, cytolysis, SARS-CoV-2, anakinra, clinical trial

## Abstract

Severe COVID-19 infection resulting in hospitalization shares features with cytokine storm syndromes (CSSs) such as hemophagocytic lymphohistiocytosis (HLH). Various published criteria were explored to define CSS among patients (n = 32) enrolled in a COVID-19 clinical trial. None of the patients met HLH-04 or HScore criteria, but the ferritin to erythrocyte sedimentation rate (ferritin–ESR) ratio and the COVID-19 cytokine storm score (CSs) identified 84% and 81% of patients, respectively. As 30–40% of patients in published secondary HLH cohorts possess rare heterozygous mutations in familial HLH (fHLH) genes, whole genome sequencing was undertaken to explore immunologic gene mutation associations among 20 patients enrolled in the trial. Rare mutations in fHLH genes were identified in 6 patients (30%), and 4 patients (20%) possessed rare mutations in *DOCK8* (a novel CSS gene). Foamy viral transduction of the 3 *DOCK8* missense mutations into NK-92 natural killer (NK) cells diminished NK cell cytolytic function, a feature of HLH. This severe COVID-19 cohort, like others, shares CSS features but is best identified by the ferritin–ESR ratio. Rare heterozygous CSS gene (fHLH genes and *DOCK8*) mutations were frequently (45%) identified in this severe COVID-19 cohort, and *DOCK8* missense mutations may contribute to CSS via diminished lymphocyte cytolytic activity.

## 1. Introduction

Cytokine storm syndromes are hyperinflammatory febrile states associated with rare genetic defects (e.g., perforin deficiency), infections (e.g., Epstein–Barr virus), rheumatic diseases (e.g., Still disease), and hematopoetic malignancies (e.g., T-cell lymphoma) [1]. CSS can result in multi-organ system failure and is frequently fatal [2]. The cytokine storm syndrome (CSS) associated with severe SARS-CoV-2 infection (COVID-19) is somewhat unique but shares features with other CSS, such as hemophagocytic lymphohistiocytosis (HLH) and macrophage activation syndrome (MAS) [3]. Different CSSs are classifiable by various published criteria [4]. These include HLH-04 and HScore criteria among others [5,6]. Neither the HLH-04 nor the HScore criteria perform well at identifying COVID-19 CSS, and during the COVID-19 pandemic, various CSS criteria were introduced to identify severe SARS-CoV-2 infection [7]. Approximately 10–15% percent of patients infected with SARS-CoV-2 early during the pandemic developed severe COVID-19, requiring hospitalization [8]. A clinical trial to treat severe SARS-CoV-2 infection with the interleukin-1 inhibitor, anakinra (beneficial for secondary HLH, sHLH) [9], was recently published [8]. Entry criteria included several features of CSS, including elevated serum C-reactive protein, ferritin, liver enzymes, and D-dimers, as well as diminished levels of lymphocytes and platelets [8]. Of 235 hospitalized severe COVID-19 patients screened, only 53 met the stringent CSS-based entry criteria, making them the sickest of the sick [8]. Herein, various published HLH and MAS criteria, as well as COVID-19-specific CSS criteria, were analyzed to explore their utility in identifying CSS in the setting of COVID-19. As previously noted [7], the HLH-04 and HScore criteria performed poorly, but the ferritin to erythrocyte sedimentation rate (ESR) ratio (ferritin–ESR) [10] and the COVID-19 cytokine storm score (CSs) [11] identified over 80% of those enrolled in this severe COVID-19 clinical trial [8]. Thus, the subset of COVID-19 patients in this trial shared features of CSS [12].

Why some individuals with various infections, including SARS-CoV-2, develop severe disease manifestations including features of CSS is unclear. However, fatal cases of another severe respiratory infection, H1N1 influenza, have been associated with heterozygous mutations in genes linked with familial HLH (fHLH) [13]. Indeed, mutations in several fHLH genes involved in perforin-mediated cytolysis by cytotoxic T-cells and natural killer (NK) cells have been identified in individuals with severe COVID-19 [14,15,16,17,18]. Mutations in novel and related sHLH-associated genes, *DOCK2* and *DOCK8*, which are important for lymphocyte cytolytic activity [19,20], have also been linked to severe COVID-19 [21] and post-COVID-19 multi-system inflammatory syndrome in children (MIS-C) [22], respectively. Moreover, poor lymphocyte cytolytic activity, in general, has also been associated with severe COVID-19 [23]. In exploring immune gene mutations by whole genome sequencing (WGS) among 20 of the severe COVID-19 patients enrolled in the anakinra clinical trial, several fHLH genes and *DOCK8* mutations were identified in the cohort. Functional testing of the *DOCK8* mutations, the most frequently mutated gene (n = 4, 20%), was performed. Missense mutations were explored in NK cells, demonstrating decreased cytolytic function. Thus, mutations in fHLH and *DOCK8* genes may serve as risk alleles for developing severe COVID-19 with features of CSS upon infection with SARS-CoV-2.

## 2. Materials and Methods

### 2.1. Study Participants

The double-blind, randomized, placebo-controlled trial of anakinra for severe COVID-19 was previously reported [8] and was registered with ClinicalTrials.gov, NCT04362111, where the full list of eligibility criteria is provided. In brief, inclusion criteria were as follows: 1. 18 years old or older; 2. molecular diagnosis of SARS-CoV-2 infection and COVID-19 pneumonia; 3. radiographic imaging consistent with COVID-19 pneumonia; 4. room air oxygen saturation <93%; 5. hyperferritinemia (>700 ng/mL); 6. any 3 of the following: a. elevated D-dimer (>500 ng/mL), b. thrombocytopenia (<130,000/mm^3^), c. leukopenia (WBC < 3500/mm^3^) or lymphopenia (<1000/mm^3^), d. elevated AST or ALT (>2X ULN), e. elevated LDH (>2X ULN), or f. CRP >100 mg/L. The University of Alabama at Birmingham (UAB) Institutional Review Board approved the trial (IRB-300005216), which was conducted in accordance with Good Clinical Practice guidelines. The study was funded by the UAB School of Medicine COVID-19 Research Initiative (to R.Q.C. and W.W.C.), and Swedish Orphan Biovitrum (Sobi) provided the anakinra and placebo but played no role in data analysis, data interpretation, or manuscript preparation.

### 2.2. Data Collection and Statistical Analysis

Clinical and laboratory variables were collected on a UAB secure server, and for purposes of CSS criteria fulfillment, only data obtained within 72 h of hospitalization were analyzed. An Excel spreadsheet was used to record and calculate the various HLH/MAS/CSS criteria for each of 32 patients enrolled in the clinical trial. The following criteria were calculated as previously described [7]: HLH-04 [24], HScore [5], sJIA MAS score [25], MS score [26], ferritin–ESR ratio [10], COVID-19-associated hyperinflammatory syndrome (cHIS criteria) [27], COVID-Cytokine Storm (COVID-CS) [28], and CSs [11]. Continuous variables are reported as medians with interquartile ranges, and categorical variables are summarized as frequencies and percentages. Comparisons of nonparametric laboratory values were made using the Kruskal–Wallis test with a significance *p* value of 0.05. Demographics, comorbidities, and other treatments are previously reported [8].

### 2.3. Whole Genome Sequencing (WGS) and Analysis

#### 2.3.1. Sequencing and Analysis Process

Whole genome sequencing (WGS) was conducted among the first 20 patients enrolled for whom samples for WGS were available using the HudsonAlpha Clinical Services Laboratory (CSL) and HudsonAlpha-Discovery. The CSL is a CAP/CLIA genomics lab that currently handles samples and, together with HudsonAlpha Discovery, generates sequencing data for all the translational genomics research projects. Sequencing libraries were constructed using genomic DNA isolated from frozen buffy coat. Blood samples were collected by health care personnel, and buffy coats were prepared by biobank personnel at the clinical site, before being provided to the investigative team. Because COVID-19 is transmitted by respiratory spread and not by blood, DNA preparation by the team does not constitute an excessive biohazard beyond the handling of any other blood or blood-derived sample, and DNA preparation was handled by existing protocols to ensure safety and compliance with all relevant requirements. Genome sequencing was completed to an average of 30X coverage using Illumina NovaSeq sequencers generating 150 bp paired-end reads. Resulting sequence reads were aligned to the reference genome (GRCh38) using DRAGEN software (v07.011.352.3.2.8b). Detection of single-nucleotide variants (SNVs) and small insertion-deletion variants (indels) was performed using GATK (v3.8), in accordance with community best practices and our typical workflow.

#### 2.3.2. Analyzing Variants to Search for Highly Penetrant Genetic Factors

Variation in each proband was analyzed using Codicem, a software platform built at HudsonAlpha to facilitate interpretation of variants in translational research projects and CSL’s clinical genomic testing. Codicem performs variant annotation and filtration with commonly used features (e.g., frequencies in gnomAD, gene body effects, conservation scores, impact predictions, genic intolerance scores, etc.). Codicem also provides automated American College of Medical Genetics and Genomics evidence code predictions for all variants and flags genes that are associated with disease in databases like Online Mendelian Inheritance in Man (OMIM). These data are presented to a variant analyst in an interactive form along with features like quality control metrics, links to gene-specific publications, information from clinical databases like ClinVar, previous in-house interpretations, and other features useful for variant curation and assessment.

Structural variants (SV) were called using four software packages, including Manta [29], Delly [30], ERDS [31], and CNVnator [32]. Mobster [33], Retroseq [34], and MELT [35] are also used to identify mobile element insertions (MEIs), including Alu, L1, and SVA events. While difficult to detect, MEIs have been identified in a number of rare disease cases [36], and the pipeline has led to the identification of three medically relevant variants (unpublished). A customized, automated pipeline then integrates the output from these callers into a single unified and annotated set of SVs suitable for filtration and curation. While some SVs can be analyzed within Codicem, many require customized tools. Towards that end, a mix of both off-the-shelf and in-house tools are used. For example, for all SVs that are of interest, the Integrative Genomics Viewer (IGV, [37]), an automated pipeline to generate images that show read-depth and SNV allelic balance metrics in the context of segmental duplications, mobile elements, gnomAD variant frequencies, and genes, similar in some ways to that seen in Cooper et al. [38] is used. Further, all SVs of interest were queried against SV databases like dbVar and against SVs found in all individuals that were previously sequenced at HudsonAlpha (over 3000). Rare and uncommon immune related gene mutations identified in the cohort are reported in Table 1.

### 2.4. DOCK8 Mutant Gene Preparations, Foamy Virus (FV) Preparation, and NK-92 Cells Infection

Wild-type (WT) human *DOCK8* cDNA was previously generated [22]. Modification of the WT plasmid cDNA by site-directed mutagenesis to match the precise COVID-19 patient-derived mutant *DOCK8* sequences was as previously described [20]. The 3 unique patient-derived mutant *DOCK8* missense cDNA were confirmed by Sanger DNA sequence analysis. A recombinant foamy virus (FV) expression system for efficient infection of large cDNA, such as *DOCK8*, has been previously described [20]. Recombinant FV preparation and infection of human NK-92 NK cells were as described [20,22]. *DOCK8*-FV infected NK-92 cells were sorted based on enhanced green fluorescent protein (EGFP) co-expression by flow cytometry (FCM) prior to experimentation [20].

### 2.5. NK-92 Cell Degranulation and Cytotoxicity Assays

For degranulation assays, WT and patient-derived *DOCK8*-FV-infected NK-92 cells and K562 erythroleukemia target cells were mixed at a 2:1 effector to target cell ratio and incubated in 5% CO_2_ at 37 °C. Simultaneously, effectors (NK-92 cells in isolation) were set up as a background degranulation control. After 1.5 h, the cells were harvested, stained with fluorescein-conjugated anti-CD56 (NK cell marker, Pacific Blue, Biolegend, San Diego, CA, USA) and anti-CD107a/LAMP1 (Allophycocyanin (APC), Biolegend) antibodies. The CD56+ NK cells were then analyzed for cell surface expression of CD107a by FCM as previously described [39] using FlowJo 10.2 software (Ashland, OR, USA).

For cytotoxicity assays, WT and patient-derived *DOCK8*-FV-infected NK-92 cells and K562 erythroleukemia target cells were mixed at a 5:1 effector to target cell ratio and incubated in 5% CO_2_ at 37 °C for 4 h. Simultaneously, K562 target cells in the absence of NK-92 effector cells were set up as a background fluorescence control. The cells were harvested, stained with fluorescein-conjugated anti-CD56 and live/dead fixable cell dead reagent (Invitrogen, Waltham, MA, USA), and analyzed for cell death by FCM as previously described [39].

## 3. Results

### 3.1. Cytokine Storm Syndrome Criteria Met by Patients

Early during the pandemic (L-strain), 32 hospitalized adults with severe COVID-19 were endrolled in a clinical trial to test the potential benefit of IL-1 blockade with anakinra [8]. The patients were selected to meet criteria reflecting severe illness with features of CSS (e.g., hyperferritinemia, coagulopathy, liver dysfunction, cytopenias, elevated C-reactive protein). As severe COVID-19 was a relatively unique CSS, various COVID-19-specific criteria were quickly established [40]. Clincal features and laboratory values obtained during the first 3 days of hospital admission were used to assess the value of the established CSS criteria, and the newly developed COVID-19 CSS criteria (Table 2).

As NK cell activity and biopsy specimens to explore hemophagocytosis were not collected, modified HLH-2004 and HScore were employed. Nonetheless, none of the 32 clinical trial participants reached the threshold scores for either the modified HLH-04 [6] nor the modified HScore [5] criteria (Table 1). The 3 criteria developed for detection of MAS (a form of sHLH/CSS) in the setting of sJIA/Still disease were also examined. A modified MS score was also employed as data as to the presence of arthritis was not available. The original 2016 sJIA/MAS classification criteria [25] detected only 19% of participants, the modified MS score [26] identified 37% of participants, and the ferritin (ng/mL) to ESR (mm/h) ratio [10] with a threshold of 21.5 (for distinguishing sJIA MAS from disease flare) identified 41% of patients (Table 3). None of the established criteria performed well at identifying CSS among this cohort.

In contrast to the ferritin to ESR ratio with a threshold of 21.5, the threshold of 11.3 distinguishes sJIA MAS/CSS from systemic infection [10]. This simple and easy to obtain ratio identified 84% of severe COVID-19 patients in this trial (Table 3). Similarly, the CSs quick score [11] specific for COVID-19 CSS identified 81% of trial participants (Table 3). In contrast, another COVID-19 CSS criteria (the Caricchio score) [28] only identified one patient among the 32 (3%) (Table 3). Finally, the cHIS hyper-inflammatory score [27] detected all but 2 trial participants (94%) with a very sensitive threshold of only 2 criteria (Table 3). While there was no statistically significant correlation between the scores of the various diagnostic/classification criteria and mortality, all study participants who died had a minimum of 4 of the cHIS Score criteria (Table 2), consistent with the COVID-19 mortality association originally identified with 2 or more criteria [27]. Lastly, 7 of the 8 COVID-19 patient who died met the ferritin to ESR ratio of 11.3 threshold (Table 2). Thus, the ferritin to ESR ratio of 11.3 was both sensitive at identifying CSS among severely ill COVID-19 patients (84%), and it identified 88% of those who died in the study.

### 3.2. Whole Genome Sequencing (WGS) of Severe COVID-19 Trial Patients

WGS was conducted among the first 20 patients enrolled for whom samples for WGS were available. These were screened for rare and uncommon immune-related gene variants, including known fHLH genes and the newly identified CSS gene, *DOCK8* [20]. Sixty percent of severe COVID-19 patients had heterozygous mutations in primary immunodeficiency genes for disorders that are largely recessive but rarely can have dominant phenotypes (Table 1, gray highlights). Fifteen percent of the cohort had heterozygous mutations in genes associated with autoinflammatory disorders but did not have underlying features of disease (Table 1, blue highlights). Likely, none of the autoimmune nor autoinflammatory mutations were disease contributory as heterozygotes.

Interestingly, 30% percent of DNA sequenced severe COVID-19 trial participants possessed novel, rare, or uncommon variants/mutations in known fHLH genes (Table 1, green highlights). Three of these 6 patients possessed 2 or 3 fHLH gene variants. In addition, 4 individuals (20% of those sequenced) had *DOCK8* gene variants/mutations (Table 1, pink highlights). Three of these rare or uncommon *DOCK8* variants/mutations were missense (amino acid changing), and one was intronically located. Various missense mutations in *DOCK8* from CSS patients have previously been shown to act as partial dominant-negative mutations disrupting NK cell cytotoxicity similar to fHLH gene mutations [20,22].

### 3.3. Functional Testing of DOCK8 Missense Mutations

**Figure 1:**
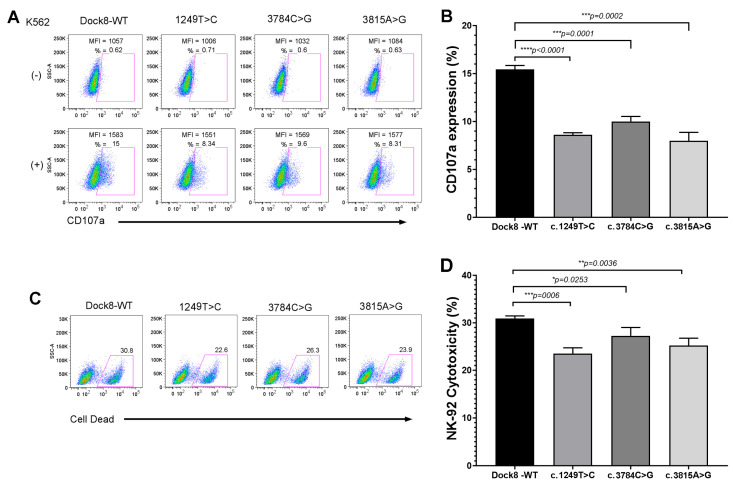
*DOCK8* missense mutations partially disrupt NK cell cytotoxicity. (**A**) Representative FCM plots (*Y*-axis: side scatter, *X*-axis: CD107a expression) of NK-92 cell degranulation following incubation without (top row) or with (bottom row) K562 target cells (from left to right, NK-92 cells with FV expression of *DOCK8* WT, c.1249T>C, c.3784C>G, c.3815A>G). Percent expression and MFI (mean fluorescence intensity are noted). (**B**) Bar graph of percent CD107a expression means ± SEM (n = 4) for NK-92 cells with FV expression of *DOCK8* WT, c.1249T>C, c.3784C>G, c.3815A>G. (**C**) Representative FCM plots (*Y*-axis: side scatter, *X*-axis: cell death reagent) of K562 (CD56 gated) cell death in the presence of NK-92 cells with FV expression of (left to right: *DOCK8* WT, c.1249T>C, c.3784C>G, c.3815A>G). (**D**) Bar graph of K562 cell death percentage means ± SEM (n = 4) by NK-92 cells with FV expression of *DOCK8* WT, c.1249T>C, c.3784C>G, c.3815A>G. * = *p* < 0.05, ** = *p* < 0.005, *** = *p* < 0.001, **** = *p* < 0.0001.

As the 3 missense *DOCK8* mutations identified in this severe COVID-19 cohort had not previously been functionally tested, cDNA for the individual *DOCK8* mutations were generated for use in FV transduction of the human NK cell line, NK-92 (see Section 2). FV transduction of NK-92 cells allowed for co-expression of WT or severe COVID-19 patient-derived *DOCK8* missense mutation along with EGFP for cell sorting. EGFP+ FV transduced NK-92 cells were incubated with K562 target cells and tested for degranulation (CD107a), a process disrupted in fHLH perforin pathway disruption. Each of the 3 *DOCK8* missense mutations independently partially disrupted (~50%) degranulation in comparison to WT *DOCK8* control (Figure 1A,B). In addition, each of the 3 *DOCK8* missense mutations independently partially disrupted NK cell cytotoxicity (~20%) as detected by FCM (Figure 1C,D). Since NK-92 cells already express WT *DOCK8*, these 3 *DOCK8* missense mutations appear to act as partial dominant-negative mutations.

Since 45% of the clinical trial participants who underwent WGS possessed mutations in fHLH and/or *DOCK8* genes that may have contributed to CSS via partially disrupted NK cell cytolysis, the association of these mutations was compared with the various CSS (general and COVID-19 specific) CSS criteria. Comparing patients with fHLH or *DOCK8* gene mutations to those without these mutations was generally non-revealing (no obvious correlation) for most criteria explored (Table 4). Nonetheless, the ferritin to ESR threshold of 11.3 identified all (9/9) severe COVID-19 patients with a *DOCK8* and/or fHLH gene mutation but also all of those without one of these mutations. Thus, the ferritin to ESR ratio may help identify severe COVID-19 patients with shared CSS pathophysiology (decreased lymphocyte cytolytic activity) to fHLH and sHLH [41,42]. This has implications for therapeutic approaches for severe COVID-19 [43,44,45].

## 4. Discussion

COVID-19 has killed over 7 million people worldwide, including over 1 million in the United States, many of whom suffered a CSS [1]. CSSs are frequently triggered by infections, yet SARS-CoV-2 (the etiology of COVID-19) severe infections invoke a relatively unique CSS that is poorly identified by pre-existing CSS criteria [3,7]. The urgency of the COVID-19 pandemic, and the lack of utility of prior CSS criteria [5,24], triggered multiple attempts to define the COVID-19 CSS [11,27,28]. Unfortunately, both the broad and COVID-19-specific CSS criteria often perform poorly [7,46].

The clinical trial of anakinra for severe COVID-19 with features of CSS studied herein [8] confirmed the poor performance of prior CSS/HLH criteria in identifying CSS among hospitalized severe COVID-19. However, the ferritin–ESR ratio with a threshold of 11.3 [10] and the cytokine storm score (CSs) [11] both identified over 80% of the severe COVID-19 patients in this clinical trial. Being so sensitive, the ferritin–ESR ratio identified severe COVID-19 patients regardless of *DOCK8* or fHLH mutation (Table 4), likely reflecting other risk factors for severe disease in those without known CSS gene mutations. The small number of patients studied is a limitation of this study, and the value of the ferritin–ESR ratio in identifying COVID-19 CSS should be validated in larger cohorts. Nevertheless, increased criteria met of the COVID-19-associated hyperinflammatory syndrome (cHIS) correlated with mortality as previously reported [27]. Common to most all CSS criteria, COVID-19 included, is an elevated serum ferritin (typically >500–700 ng/mL), and as it is a simple, inexpensive, timely, and widely available laboratory, it can be used as a valuable sensitive tool for helping to identify febrile hospitalized individuals with probable CSS [47,48].

The ferritin–ESR ratio has previously been used to screen for MAS in the setting of Still disease [10,49]. In distinguishing between Still disease MAS and hospitalized febrile controls, the ferritin–ESR ratio demonstrated 78% specificity at a sensitivity of 95% [10]. Simplistically explained, in the setting of MAS, the ferritin rises with inflammation, and the ESR drops when coagulopathy ensues from diminished fibrinogen [10]. Severe COVID-19 also shares hyper-inflammation, but the relative rise in ferritin is typically less than seen in MAS [3]. However, with SARS-CoV-2 infection, the extent of thrombosis is considerably higher than in other MASs, as demonstrated by the frequently noted elevations of D-dimer during this infection [50]. There is therefore an attendant relative decrease in plasma fibrinogen, which would otherwise be elevated and promote red blood cell (RBC) clumping via neutralization of the positive RBC membrane charge that otherwise repels erythrocyte self-aggregation. Absent this impact of elevated intact fibrinogen on the speed of RBC sedimentation, the elevation of plasma proteins during the acute stages of the viral infection increases plasma viscosity, resulting in ESR levels that are lower than one might expect for a highly inflammatory state [51]. Thus, the ESR –ferritin ratio is a sensitive tool for identifying severe COVID-19 infection.

In addition to hyperferritinemia being common to many forms of CSS, heterozygous missense mutations in fHLH genes are seen in ~30–40% of many published CSS/sHLH cohorts [9,42,52]. The contribution of the individual fHLH gene mutations to a threshold model of CSS disease [53], however, remains unclear. Functionally testing the specific fHLH gene mutations in vitro or ex vivo may lead credence to causality [13,39,42,54,55], but even this approach raises challenges [56].

Recently, a novel CSS gene, *DOCK8*, has been proposed to be associated with CSS/sHLH [20]. *DOCK8* missense mutations have been shown to diminish lymphocyte cytolytic activity in CSS patients with infection triggered CSS, autoimmune triggered CSS, as well as the post-COVID-19 CSS, MIS-C [20,22]. It is quite remarkable that 20% of the severe COVID-19 patients sequenced from this clinical trial [8] possessed *DOCK8* mutations (Table 1), and 10% (4/39) of MIS-C children who underwent DNA sequencing had *DOCK8* mutations [22]. Functionally, all 7 of these *DOCK8* missense mutations acted in a partial dominant-negative fashion (Figure 1) [22]. It is possible the *DOCK8* intronic mutation (Table 1, patient 10) results in diminished DOCK8 activity, similar to an earlier reported intronic mutation in the fHLH gene, *UNC13D* [55]. Previously, a homozygous *DOCK8* defect [57] and a heterozygous intronic splice site *DOCK8* mutation that disrupted mRNA splicing [20] were reported in children with CSS.

DOCK8, and the related protein, DOCK2, possess GTPase activity important for actin cytoskeleton function, including trafficking of perforin-containing cytolytic granules to the immunologic synapse, and DOCK2 and DOCK8 proteins are also required for optimal synapse formation to allow for effective perforin-mediated lysis of target cells [20,58,59,60,61]. Defects in perforin-mediated NK cell and CD8 T cell cytolysis in the setting of infection result in prolonged engagement of the lytic lymphocyte and its infected target cell (antigen presenting cell) [62,63]. This prolonged engagement yields excess pro-inflammatory cytokines responsible for the multi-organ system failure in CSS [39,62,63]. Presumably, disruptive *DOCK8* mutations contribute to CSS in severe COVID-19 as well [12,41,44,64]. Along these lines a *DOCK8* mutation was reported among a cohort of 8 fatal cases of COVID-19 with CSS [65]. Thus, *DOCK8* may be a risk allele for COVID-19 CSS development.

Whether or not *DOCK8* is a risk allele for COVID-19 infection cannot be ascertained from this current study and is a limitation, as non-severe COVID-19 patients did not undergo WGS. It seems unlikely, however, that *DOCK8* gene mutations are as frequent as in the severe COVID-19 cohort in this study. It is striking that 20% of the severe COVID-19 patients possessed rare or uncommon variants with functional impacts on NK cell function. Based on the frequency of the most common of these variants (0.27%), one would expect less than one in 350 individuals to possess a similar *DOCK8* mutation. Nonetheless, the small sample size of 20 severe COVID-19 patients who underwent CSS is a study limitation, and future studies are needed to replicate the role of *DOCK8* in severe COVID-19.

Like *DOCK8*, *DOCK2* mutations have also recently been reported in association with CSS [19,66], and *DOCK2* has been implicated in severe COVID-19 pneumonia [21,67]. Homozygous and hemizygous *DOCK11* deficiency has also been newly associated with hyper-inflammatory states [68,69], but whether *DOCK11* mutations contribute to CSS or severe COVID-19 is currently unknown. For now, further exploration of the association and role of fHLH genes and *DOCK8* in severe COVID-19 CSS is worthy of exploration.

## 5. Conclusions

Severe COVID-19 presents as a relatively unique CSS, but it is identifiable by a ferritin to ESR ratio >11.3, or by the Cytokine Storm score (CSs). In this severe COVID-19 cohort, 45% (9/20) of those undergoing WGS possessed heterozygous mutations in fHLH and/or *DOCK8* genes. The 3 unique *DOCK8* missense mutations identified in this cohort, similar to fHLH gene mutations, functioned to partially disrupt NK cell cytolytic function. These data suggest that fHLH and *DOCK8* gene mutations are risk alleles for development of severe COVID-19 CSS.

## 6. Patents

“Long noncoding RNA risk factor for COVID-19”, US Application No. 63/220,253, University of Alabama at Birmingham, was filed in March 2021.

## Figures and Tables

**Table 1:** Patient-derived immune-related gene variants identified by WGS.

Pt.#	Gene ^1^	Gene Description	Mutation	Frequency (gnomAD)
3	*IFNGR1*	IFN-gamma receptor 1	c.58G>A (p.Val20Ile)	0.27%
	*LYST*	Chediak-Higashi (fHLH)	c.3683A>G (p.Asn1228Ser)	0.17%
	*NLRC4*	Enterocolitis autoinflammatory	c.787+102T>C (intron)	0.38%
	*NLRP12*	Cold autoinflammatory 2	c.1063 (p.Glu355Lys)	0.16%
4	*DOCK8*	Hyper-IgE syndrome (NK function)	c.3784C>G (p.Leu1262Val)	0.09%
	*LACC1*	Oxidoreductase (systemic JIA)	c.-239+12_-239+13ins (5’ UTR)	Novel
	*NLRP4*	Pathogenic E. coli infections	c.2119_2122deltCTC (p.Ser707Valfs)	Novel
	*RAG1*	SCID (immunodeficiency)	c.295G>A (p.Gly99Ser)	0.09%
6	*C7*	Complement 7	c.1561C>A (p.Arg521Ser)	0.25%
	*CARMIL2*	Combined immunodeficiency	c.2489G>A (p.Arg830Gln)	0.56%
	*TNFAIP3*	Behcet-like autoinflammatory	c.755A>G (p.Tyr252Cys)	0.0007%
10	*DOCK8*	Hyper-IgE syndrome (NK function)	c.53+199C>G (intron)	0.21%
	*MRTFA*	Immunodeficiency 66 (TF)	c.1693dupG (p.Ala565GlyfsTer67)	Novel
11				
12	*DOCK8*	Hyper-IgE syndrome (NK function)	c.1249T>C (p.Phe417Leu)	0.001%
	*IL17RA*	Immunodeficiency 51 (IL-17 rec. A)	c.1361 (p.Pro454Arg)	0.001%
	*ITK*	IL-2 induc. T cell kinase (agamma.)	c.1510A>T (p.Thr504Ser)	0.09%
	*LRBA*	Common variable immunodef. 8	c.7708+337C>T (intron)	0.12%
	*XIAP*	X-linked lymphoproliferative 2	c.1408A>T (p.Thr470Ser)	0.05%
13	*LYST*	Chediak-Higashi (fHLH)	c.891T>G (p.ASn2971Lys)	0.27%
	*STAT2*	Immunodeficiency 44	c.2378A>T (p.His793Leu)	Novel
	*STXBP2*	UNC18-2 (fHLH)	n.1610G>C (non-coding exon)	0.14%
14	*IFNGR1*	IFN-gamma receptor 1	c.1325A>G (p.Glu442Gly)	Novel
15	*LYST*	Chediak-Higashi (fHLH)	c.1889C>T (p.Pro630Leu)	Novel
	*LYST*	Chediak-Higashi (fHLH)	c.3359G>T (p.Ser1120Ile)	0.29%
	*NLRC4*	Enterocolitis autoinflammatory	c.262+1187G>A (intron)	0.14%
	*RAB27A*	Griscelli 2 syndrome (fHLH)	c.468-3C>T (3’ splice site)	0.23%
	*STAT3*	Hyper-IgE syndrome (TF)	c.550+250C>A (intron)	1.7%
16	*DOCK8*	Hyper-IgE syndrome (NK function)	c.3815A>G (p.Tyr1272Cys)	0.27%
17				
18	*STXBP2*	UNC18-2 (fHLH)	c.1529+10C>T (intron)	0.21%
20	*WAS*	Wiskott-Aldrich syndrome	c995T>C (p.Val332Ala)	0.49%
21				
22	*LRBA*	Common variable immunodef. 8	c.5291+3024A>G (interior intron)	0.019%
	*PIK3CD*	PI kinase catalytic	c.*270C>T (3’ of stop codon)	0.081%
23				
24				
25	*CASP10*	ALPS lymphoproliferative syndrome	c.*1043C>T (3’ coding exon)	0.35%
	*STAT4*	SLE 11 (IL-23 signaling)	c.719G>A (p.Arg240Gln)	0.14%
	*UNC13D*	MUNC13-4 (fHLH)	c.2542A>C (p.Ile848Leu)	0.10%
	*UNC13D*	MUNC13-4 (fHLH)	c.2983G>C (p.Ala995Pro)	0.10%
26	*MRTFA*	Immunodeficiency 66 (TF)	c.1603G>A (p.Glu535Lys)	0.025%
27				

^1^ fHLH gene: 30%; *DOCK8* novel HLH gene: 20%; autoinflammatory: 15%; immunodeficiency:60%.

**Table 2:** Established and COVID-19-specific cytokine storm syndrome criteria met by the clinical trial participants.

Patient	Placebo (P), Anakinra (A), or Withdraw (WD)	* HLH-2004 Score (≥5)	** HScore (≥169)	2016 sJIA/MAS Score	*** MS Score (≥−2.1)	Ferritin–ESR (>11.3)	CSs Score	cHIS Score (≥2)	Caricchio Score
1	A	2	87	No	−2.2	19	Pos	2	No
2	A	2	107	No	−2.5	16	Pos	3	No
3	P	2	83	No	−3.0	19.3	Pos	5	No
4	**P**	**3**	**83**	**No**	**−1.5**	**72.3**	**Pos**	**4**	**No**
5	WD	3	83	Yes	−2.5	9.1	Pos	3	No
6	**P**	**3**	**157**	**Yes**	**4.5**	**16.1**	**Neg**	**5**	**Yes**
7	A	1	63	No	−2.4	15.7	Pos	3	No
8	**A**	**3**	**118**	**Yes**	**1.5**	**40.4**	**Pos**	**5**	**No**
9	WD	3	83	No	−3.6	11.6	Pos	3	No
10	A	1	63	No	−0.1	15.9	Pos	3	No
11	**A**	**3**	**97**	**Yes**	**−2.8**	**28.6**	**Pos**	**5**	**No**
12	P	2	63	No	−3.8	32.2	Neg	2	No
13	A	2	107	No	−2.6	18.31	Pos	3	No
14	P	1	44	No	−2.1	12.8	Pos	3	No
15	**P**	**1**	**64**	**No**	**−3.8**	**11.6**	**Pos**	**4**	**No**
16	P	1	83	No	−3.0	16.2	Neg	1	No
17	A	3	117	Yes	−1.2	63.7	Pos	2	No
18	A	2	83	No	−2.7	27	Pos	4	No
19	A	1	83	No	1.1	5.7	Neg	1	No
20	P	1	83	No	−4.2	18.8	Pos	3	No
21	P	3	118	No	−2.8	38.1	Pos	4	No
22	A	1	64	No	−1.7	99.2	Pos	3	No
23	P	1	118	No	−0.2	149.8	Pos	4	No
24	P	2	83	No	−2.6	79.7	Neg	2	No
25	**A**	**2**	**118**	**No**	**−1.5**	**111.8**	**Pos**	**4**	**No**
26	P	2	64	No	−2.6	12.9	Pos	2	No
27	A	1	83	No	−2.6	23.8	Pos	3	No
28	A	2	83	No	−3.2	7.9	Pos	3	No
29	**A**	**2**	**83**	**Yes**	**−3.7**	**14.4**	**Pos**	**4**	**No**
30	P	2	64	No	−1.3	24	Neg	3	No
31	P	3	83	No	−2.3	10	Pos	4	No
32	**P**	**2**	**64**	**No**	**−0.7**	**8.6**	**Pos**	**4**	**No**

* Modified HLH-2004 Criteria—hemophagocytosis and NK cell cytolytic data not collected; ** Modified HScore Criteria—hemophagocytosis data not collected; *** Modified MS Score Criteria—Arthritis status not collected. Abbreviations: HLH-2004 = hemophagocytic lymphohistiocytosis; HScore = Hemophagocytic Syndrome; 2016 sJIA Score = systemic juvenile idiopathic arthritis; MS Score = Macrophage activation syndrome/systemic juvenile idiopathic arthritis; CSs Score = COVID-19 CSS Quick Score; cHIS Score = COVID-19-associated hyperinflammatory syndrome. Red text indicates deceased patient following anakinra trial for COVID-19 induced cytokine storm.

**Table 3:** Summary and performance of various CSS criteria in identifying CSS among the COVID-19 cohort.

	Total Patients (n = 32)
2016 sJIA/MAS Score: Yes	6/32 (19%)
MS Score, Median (IQR)	−2.5 (−3.0 to −1.4) (37%)
Ferritin–ESR > 11.3	27/32 (84%)
Ferritin–ESR > 21.5	13/32 (41%)
Ferritin–ESR, Median (IQR)	18.6 (12.8 to 36.6)
CSs Score: Pos	26/32 (81%)
cHIS Score, Median (IQR)	3 (3 to 4) (94%)
Caricchio Score	1/32 (3.1%)

Abbreviations: sJIA = systemic juvenile idiopathic arthritis; MAS = macrophage activation syndrome; MS = MAS/sJIA; ESR = erythrocyte sedimentation rate; IQR = interquartile range; CSs = cytokine storm score; cHIS = COVID-19-associated hyperinflammatory syndrome.

**Table 4:** Comparison of CSS criteria percentages met by severe COVID-19 patients with or without *DOCK8* and/or fHLH gene mutations.

	**Non-Mutant** **(n = 11)**	***DOCK8*** **(n = 4)**	**fHLH** **(n = 6)**
2016 sJIA/MAS Score Yes	3/11 (27%)	0/4 (0%)	0/6 (0%)
MS Score, Median (IQR)	−2.6 (−2.8 to −1.2)	−2.3 (−3.6 to −0.5)	−3 (−3.3 to −1.8)
Ferritin–ESR > 11.3	11/11 (100%)	4/4 (100%)	6/6 (100%)
Ferritin–ESR > 21.5	7/11 (64%)	2/4 (50%)	3/6 (50%)
Ferritin–ESR, Median (IQR)	28.6 (18.1 to 79.7)	24.2 (16 to 62.3)	23.1 (16.6 to 52.1)
CSs Score Pos	9/11 (82%)	2/4 (50%)	5/6 (83.3%)
cHIS Score, Median (IQR)	3 (2 to 4)	2.5 (1.3 to 3.8)	4 (2.8 to 4.3)
Caricchio Score	1/11 (9%)	0/4 (0%)	0/6 (0%)

Abbreviations: fHLH = familial hemophagocytic lymphohistiocytois sJIA = systemic juvenile idiopathic arthritis; MAS = macrophage activation syndrome; MS = MAS/sJIA; ESR = erythrocyte sedimentation rate; IQR = interquartile range; CSs = cytokine storm score; cHIS = COVID-19-associated hyperinflammatory syndrome.

## Data Availability

The data underlying this article cannot be shared publicly for the privacy of individuals that participated in the clinical trial. De-identified data will be shared on reasonable request to the corresponding author.
